# The impact of inspiratory pressure on stroke volume variation and the evaluation of indexing stroke volume variation to inspiratory pressure under various preload conditions in experimental animals

**DOI:** 10.1007/s00540-015-1995-y

**Published:** 2015-03-15

**Authors:** Yu Kawazoe, Tsuyoshi Nakashima, Toshie Iseri, Chiaki Yonetani, Kentaro Ueda, Yuka Fujimoto, Seiya Kato

**Affiliations:** 1 Department of Emergency and Critical Care Medicine, Wakayama Medical University, 811-1, Kimiidera, Wakayama, 641-8510 Japan; 2 Department of Advance Clinical Medicine, Graduate School of Life and Environmental Sciences, Osaka Prefecture University, 1-58, Rinku Ohrai Kita, Izumisano, Osaka 598-8531 Japan

**Keywords:** Fluid responsiveness, Inspiratory pressure, Preload, Stroke volume, Tidal volume

## Abstract

**Purpose:**

Stroke volume variation (SVV) measures fluid responsiveness, enabling optimal fluid management under positive pressure ventilation. We aimed to investigate the effect of peak inspiratory pressure (PIP) on SVV under various preload conditions in experimental animals and to ascertain whether SVV indexed to PIP decreases the effect.

**Methods:**

Mild and moderate hemorrhage models were created in nine anesthetized, mechanically ventilated beagle dogs by sequentially removing 10 and then an additional 10 ml/kg of blood, respectively. In all the animals, PIP was incrementally increased by 4 cmH_2_O, from 5 to 21 cmH_2_O. SVV was measured by arterial pulse contour analysis. Stroke volume was derived using a thermodilution method, and central venous pressure and mean arterial pressure were also measured.

**Results:**

SVV increased according to PIP with significant correlation at baseline, with mild hemorrhage and moderate hemorrhage. PIP regression coefficients at baseline and in the mild and moderate hemorrhage models were 0.59, 0.86, and 1.4, respectively. Two-way repeated-measures analysis of variance showed that PIP and the degree of hemorrhage had a significant interaction effect on SVV (*p* = 0.0016). SVV indexed to PIP reflected the hemorrhage status regardless of PIP changes ≥9 cmH_2_O.

**Conclusions:**

PIP is significantly correlated with SVV, even under hypovolemia, and the effect is enhanced with decreasing preload volumes. Compared with SVV, the indexed SVV was less susceptible to higher inspiratory pressures.

## Introduction

Stroke volume variation (SVV) is a hemodynamic parameter derived from arterial pulse contour analysis that reflects the respiratory changes in stroke volume (SV) under positive pressure ventilation. This widely used indicator is clinically applied in emergency departments, intensive care units, and operating rooms. The accuracy of SVV-based measurements of fluid responsiveness at tidal volumes (Vt) of 8–10 ml/kg has been evaluated previously [1–4]. These studies showed that an increased SVV reflected a hypovolemic state and could be used as a sensitive indicator of fluid responsiveness. However, regarding current critical care, particularly in patients with acute respiratory distress syndrome (ARDS), ventilation using such a high Vt is not recommended. Instead, protective lung strategies using low Vt and high positive-end expiratory pressures (PEEP) are generally adopted [5, 6]. In contrast, high Vt ventilation (15–20 ml/kg) with no PEEP is used in patients with cervical spinal cord injury [7, 8]. Thus, it is important to understand how SVV varies with different ventilatory settings under different preload conditions and to make appropriate adjustments for the prediction accuracy of SVV.

Vistisen et al. [9–11] reported that indexed dynamic parameters, which are acquired by dividing the parameter by Vt, improve the accuracy for prediction of fluid responsiveness at lower Vt. We aimed to investigate the effects of peak inspiratory pressure (PIP) on SVV under various preload conditions, and to verify whether the SVV values indexed to PIP reduce the confounding effects caused by variation of the inspiratory pressure.

## Methods

The present study was performed following The Science Council of Japan guidelines for animal experimentation after approval from the ethics committee for Animal Experimentation of Osaka Prefecture University, Japan.

Nine beagle dogs weighing approximately 10–12 kg were evaluated. A cannula was inserted into a peripheral vein, and butorphanol tartrate was continuously administered at a rate of 0.1 mg/kg/h. Following the subcutaneous injection of 0.025 mg/kg atropine, 0.5 mg/kg diazepam was injected intravenously during preoxygenation. Propofol was continuously injected at a rate of 8–16 mg/kg/h, and the animals were intubated with a cuffed endotracheal tube, with an internal diameter of 6.0–7.0 mm, once anesthesia was established. To enable controlled mechanical ventilation, spontaneous respiration was suspended by administration of a 1.0 mg/kg bolus of rocuronium bromide with train-of-four monitoring. Mechanical ventilation was performed using the pressure control mode with 50 % oxygen, a PIP of 5–7 cmH_2_O, an inspiration to expiration ratio of 1:2, and a respiratory rate of 20 breaths/min (Evita 4, Dräger Medical, Lübeck, Germany). PEEP was not applied to avoid its confounding effects on hemodynamics. During preparation, end-tidal CO_2_ was adjusted to within 35–45 mmHg by changing the respiratory rate. Arterial pressure was measured continuously using a cannula inserted into the tarsal artery. SVV was measured using the Vigileo-FloTrac™ system (Edwards Lifesciences, Irvine, CA, USA) based on an arterial pulse contour analysis. The animals’ age, converted to human terms, and body surface area according to the conversion table by Nelson et al. [12] were inputted during the system set up. A thermodilution catheter (132F5, Edwards Lifesciences, Irvine, CA, USA) was inserted through an introducer (RR-A60G10S, TERUMO, Tokyo, Japan) into the right internal jugular vein to obtain continuous data of central venous pressure (CVP) and intermittent data of cardiac output derived by the thermodilution method (COtd). Following anesthesia induction, 10 ml/kg hydroxyethyl starch was administered, as needed, in order to maintain the mean arterial pressure (MAP) >60 mmHg and pulse rate within 100 beats/min, with supplementation for dehydration at baseline.

Next, PIP was incrementally increased by 4 cmH_2_O, from 5 to 21 cmH_2_O, with observation for at least 2 min between steps. Baseline measurements of SVV, CVP, MAP, COtd, heart rate (HR), and Vt were recorded at each PIP, following a stabilization period of at least 2 min. The CVP, MAP, and HR were obtained from a patient monitor (BP-608 Evolution II, Omron Colin, Tokyo, Japan). We used the thermodilution method for COtd measurements, injecting 5 ml of saline at a temperature of <8 °C through the central vein. This procedure was performed three times at PIPs of 5, 13, and 21 cmH_2_O, to avoid fluid loading.

The hemorrhage models were prepared by withdrawing blood via an introducer catheter in two steps, first removing 10 ml/kg of blood (mild hemorrhage model), followed by removal of an additional 10 ml/kg (moderate hemorrhage model). Measurements were repeated at the five PIPs under the two hemorrhage conditions using the same methods as those used during PIP changes at baseline. The withdrawn blood, which was temporarily stored in a blood bag during these protocols, was carefully readministered at the end of the experiment. SV derived from a thermodilution method (SVtd) was calculated using the formula: SVtd = COtd/HR × 1,000 (ml). SVV was also indexed to PIP and Vt.

The data were analyzed using the JMP9 software program for Windows (SAS Institute Inc., Cary, NC, USA). Correlations between two variables were analyzed using a linear regression model based on the least-squares method. Dunnett’s test was used to compare the values under hemorrhage with those at baseline, two-way repeated measures analysis of variance (ANOVA) was used to analyze the effect of the relationship between PIP and hemorrhage on SVV, and the Tukey–Kramer test was used to analyze the indexed SVV disparity at other PIPs. Differences were considered significant for *p* values <0.05. In the tables, all data are presented as the means and 95 % confidence intervals.

## Results

The dogs’ body weight ranged 10.3–12.7 kg. All hemodynamic data recorded during the study are presented in Table 1.

**Table 1:** Changes in the measured parameters in relation to inspiratory pressure and blood withdrawal

Peak inspiratory pressure (cmH_2_O)	5	9	13	17	21
Vt/w (ml/kg)^a^	7.09 [6.43, 7.74]	14.9 [13.7, 16.1]	23.8 [21.8, 25.9]	31.9 [29.4, 34.4]	40.3 [37.4, 43.2]
Baseline					
SVV (%)^a^	6.24 [5.08, 7.40]	8.23 [7.29, 9.16]	11.0 [9.53, 12.5]	13.5 [11.6, 15.3]	15.4 [13.7, 17.1]
SVtd (ml)	22.4 [16.7, 28.2]		24.0 [17.3, 30.8]		23.7 [16.2, 31.2]
COtd (l/min)	1.97 [1.54, 2.40]		2.26 [1.59, 2.92]		2.3 [1.70, 3.03]
HR (beats/min)^a^	88.8 [81.3, 96.3]		93.3 [88.2, 98.4]		102 [91.4, 113]
CVP (mmHg)	3.22 [2.15, 4.29]	3.00 [2.14, 3.86]	3.33 [2.39, 4.27]	3.22 [2.02, 4.42]	3.78 [3.14, 4.42]
MAP (mmHg)	69.8 [63.2, 76.6]	67.9 [64.1, 71.7]	69.6 [64.1, 75.0]	71.4 [66.7, 76.2]	70.6 [65.5, 75.7]
Mild hemorrhage					
SVV (%)^a^	6.69 [5.44, 7.94]	9.42 [8.11, 10.7]	13.9 [11.3, 16.6]	16.6 [12.6, 20.6]	20.3 [14.9, 25.7]
SVtd (ml)	18.9 [12.2, 25.5]		19.3 [12.1, 26.5]		18.6 [1.61, 3.51]
COtd (l/min)	1.76 [1.23, 2.28]		1.88 [1.31, 2.44]		1.94 [1.37, 2.52]
HR (beats/min)^a^	96.3 [83.8, 109]		101 [89.3, 113]		109 [93.7, 125]
CVP (mmHg)	1.22^b^ [0.710, 1.73]	1.44^b^ [0.89, 2.00]	1.78^b^ [1.27, 2.29]	1.89^b^ [1.29, 2.49]	2.56^b^ [1.61, 3.51]
MAP (mmHg)	68.8 [61.1, 76.5]	67.1 [60.4, 73.8]	72.4 [66.6, 78.3]	71.7 [64.2, 79.1]	71.2 [66.6, 75.8]
Moderate hemorrhage					
SVV (%)^a^	9.28^b^ [8.44, 10.1]	12.9^b^ [10.5, 15.4]	17.9^b^ [14.0, 21.7]	24.2^b^ [17.2, 31.3]	31.6^b^ [21.6, 41.7]
SVtd (ml)	10.4^b^ [7.21, 13.7]		10.7^b^ [7.59, 13.7]		11.0^b^ [7.76, 14.3]
COtd (l/min)	1.36 [0.96, 1.76]		1.39^b^ [0.985, 1.79]		1.53 [1.05, 2.02]
HR (beats/min)^a^	133^b^ [115, 150]		132^b^ [119, 145]		140^b^ [124, 156]
CVP (mmHg)^a^	0.778^b^ [0.137, 1.42]	0.889^b^ [0.427, 1.35]	1.11^b^ [0.649, 1.57]	1.33^b^ [0.668, 2.00]	1.78^b^ [0.938, 2.62]
MAP (mmHg)	54.0^b^ [47.6, 60.4]	57.4^b^ [50.5, 64.4]	60.7^b^ [55.1, 66.2]	60.6^b^ [52.6, 68.5]	58.3^b^ [52.0, 64.7]

Data are expressed as mean (95 % confidence interval)

^a^Significant correlation with inspiratory pressure, *p* < 0.05

^b^Significant difference in the shift from baseline to the hemorrhage model, *p* < 0.05 (Dunnett’s test)

*Vt/w* tidal volume per kg body weight, *SVV* stroke volume variation, *SVtd* stroke volume derived using a thermodilution method, *COtd* cardiac output derived using a thermodilution method, *HR* heart rate, *CVP* central venous pressure, *MAP* mean arterial pressure

### Effect of PIP on hemodynamic parameters at each preload condition

**Fig. 1:**
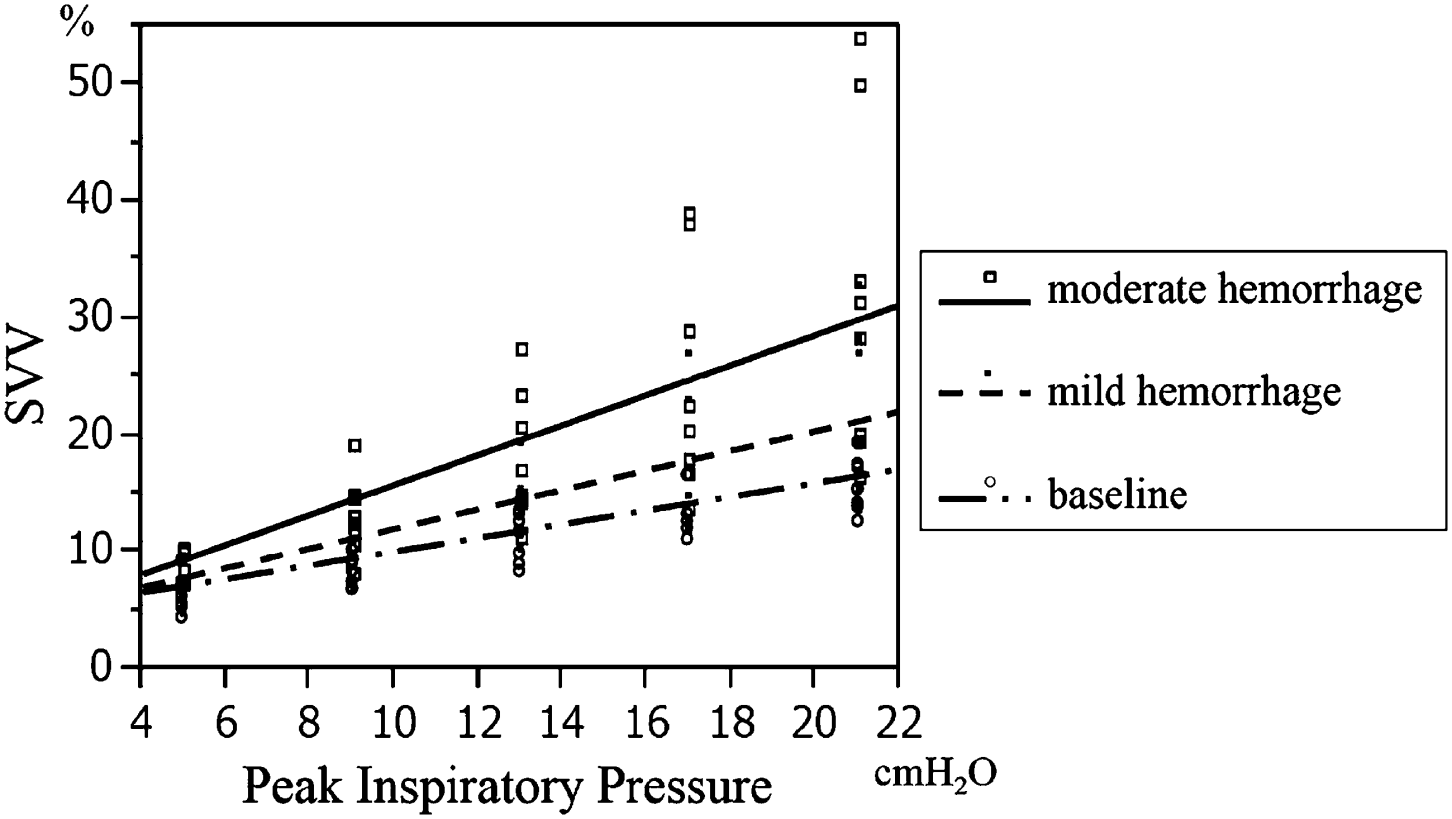
Correlation between stroke volume variation (SVV) and peak inspiratory pressure (PIP). There was a significant correlation between SVV and peak inspiratory pressure. The regression coefficients at baseline, with mild hemorrhage, and moderate hemorrhage, were 0.59, 0.86, and 1.4, respectively, indicating that the influence of PIP on SVV was enhanced by hemorrhage

Vt and PIP were significantly correlated at all time (*p* < 0.01, *R* ^2^ = 0.84). In addition, SVV was significantly correlated with PIP and Vt at each preload condition (Table 1). The regression coefficients between SVV and PIP at baseline, with mild hemorrhage, and moderate hemorrhage were 0.59, 0.86, and 1.4, respectively (Fig. 1).

Although at baseline there was no correlation between CVP and PIP (*p* = 0.31), CVP increased according to PIP in both hemorrhage models (*p* < 0.01, each) (Table 1). MAP and SVtd, calculated based on the COtd, and HR were not affected by PIP.

### Changes in hemodynamic parameters in association with changes of preload

**Fig. 2:**
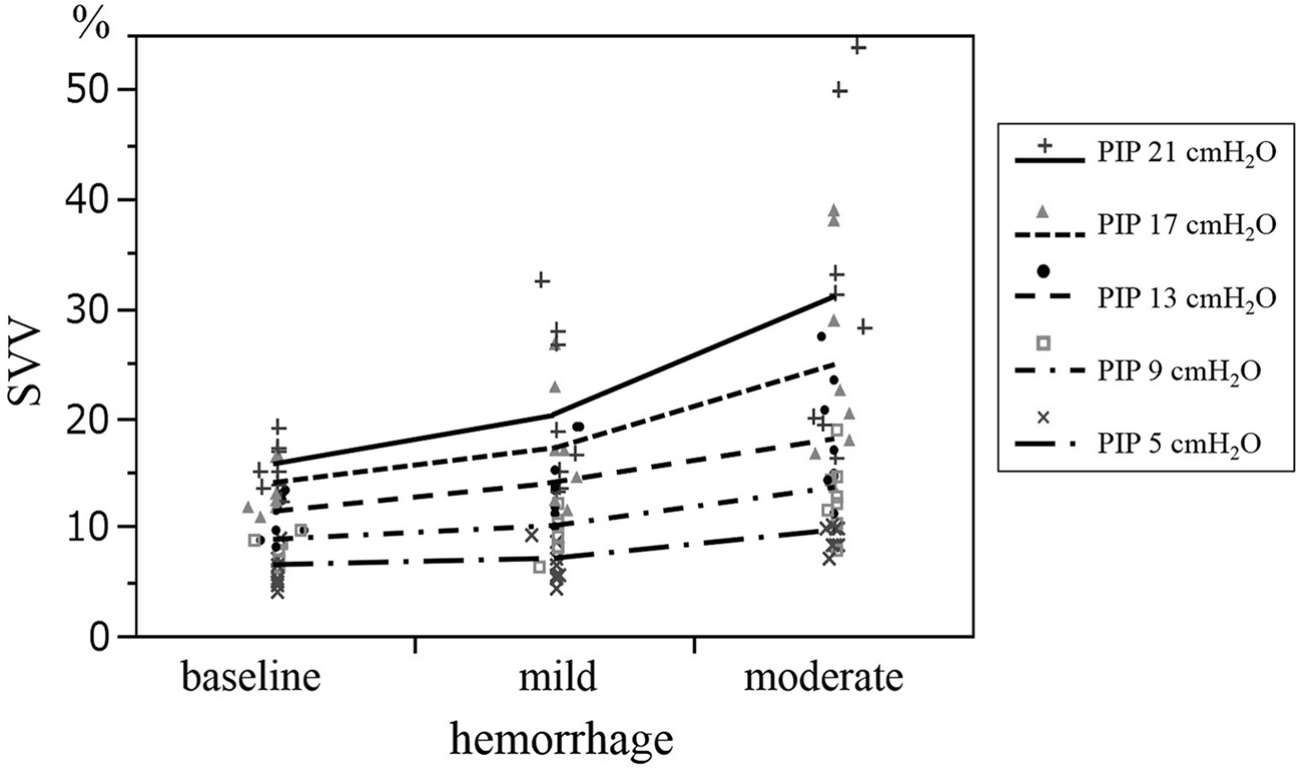
Effects of hemorrhage on stroke volume variation (SVV) at each peak inspiratory pressure (PIP). According to two-way repeated-measures analysis of variance, high PIP and hemorrhage significantly increased SVV (*p* < 0.01, each); moreover, there was a significant interaction between the effects of PIP and hemorrhage on SVV (*p* = 0.016)

All of the parameters, except for COtd, were significantly affected by moderate hemorrhage at each PIP. However, COtd was significantly decreased at a 13 cmH_2_O PIP, but not at the other PIPs. Only CVP decreased with mild hemorrhage (Table 1). Two-way repeated-measures ANOVA demonstrated that increases in PIP and hemorrhage level significantly increased SVV (*p* < 0.01, each). Moreover, there was a significantly positive interaction between the effects of hemorrhage and PIP on SVV (*p* = 0.016) (Fig. 2).

### Evaluation of SVV indexed to PIP

Table 2 shows the variations in the indexed SVV. The measured SVV was indexed to PIP and Vt. The upward trend in SVV with increasing PIP was eliminated by indexation, although there was a significant difference between the indexed SVV at 5 cmH_2_O of PIP and at the other PIPs in the same hemorrhage model, as assessed by the Tukey–Kramer test; the same relationship was noted for Vt. Similar to the SVV, the indexed SVV was increased by moderate hemorrhage.

**Table 2:** Changes in stroke volume variation indexed to inspiratory pressure and tidal volume in relation to inspiratory pressure and blood withdrawal

IP (cmH_2_O)	Indexed SVV				
	5	9	13	17	21
SVV/IP (%/cmH_2_O)					
Baseline	1.25^a^ [1.02, 1.48]	0.914 [0.810, 1.02]	0.853 [0.737, 0.969]	0.792 [0.684, 0.899]	0.733 [0.651, 0.815]
Mild hemorrhage	1.34^a^ [1.09, 1.59]	1.05 [0.901, 1.19]	1.07 [0.869, 1.27]	0.976 [0.743, 1.21]	0.967 [0.710, 1.22]
Moderate hemorrhage	1.86^a,b^ [1.69, 2.02]	1.44^b^ [1.17, 1.71]	1.37^b^ [1.08, 1.67]	1.43^b^ [1.01, 1.84]	1.51^b^ [1.03, 1.98]
SVV/Vt (%/l)					
Baseline	77.5^a^ [69.5, 85.4]	49.8 [41.8, 57.7]	41.2 [33.2, 49.1]	37.3 [29.3, 45.2]	33.8 [25.8, 41.7]
Mild hemorrhage	83.3^a^ [72.4, 94.2]	55.8 [44.9, 66.7]	52.4 [41.5, 63.3]	46.6 [35.8, 57.5]	45.1 [34.2, 55.9]
Moderate hemorrhage	125^a,b^ [106, 144]	79.6^b^ [60.4, 98.8]	69.1^b^ [49.8, 88.3]	69.5^b^ [50.3, 88.8]	71.9^b^ [52.3, 91.1]

Data are expressed as mean (95 % confidence interval)

*SVV* stroke volume variation, *IP* inspiratory pressure, *SVV/IP* stroke volume variation indexed to inspiratory pressure, *SVV/Vt* stroke volume variation indexed to tidal volume

^a^Significant difference within the same preload series, *p* < 0.05 (Tukey–Kramer test)

^b^Significant difference in the shift from baseline to the hemorrhage model, *p* < 0.05 (Dunnett’s test)

## Discussion

This is the first published study statistically demonstrating the relationship between PIP and SVV under hypovolemia and elucidating the mechanism of the interaction between PIP and preload on SVV. We made three main observations, as follows. First, SVV correlates with PIP, a determinant of Vt, even under hypovolemic conditions without any intravascular volume change. Second, there is a significant interactive effect between the relationship of PIP and SVV and that of preload and SVV, i.e., the effect of PIP on SVV is enhanced by hypovolemia. Third, SVV indexed to PIP reduces the effect of a variation of inspiratory pressure on SVV when the PIP is high enough.

Reuter et al. [13] and Szold et al. [14] demonstrated that SVV was significantly correlated with the magnitude of Vt. Moreover, Kang et al. [15] reported that SVV was elevated by increased PIP in pediatric patients. Therefore, as Lansdorp et al. [16] indicated, the predictive value of SVV is reduced in routine clinical practice because of the lower Vt used. However, to our knowledge, there are no reports regarding how the relationship between Vt and SVV changes with hypovolemia. Protective lung strategies using low Vt ventilation of 6 ml/kg and high PEEP have been recommended for the treatment of ARDS [17, 18]. The effects of this strategy on SVV have yet to be determined in detail, and we are planning a future study on the effects of inspiratory time, mean airway pressure, and PEEP on SVV. Alveolar compliance is low in patients with ARDS [19]; therefore, they require a higher ventilatory pressure to maintain the prefixed Vt, suggesting the need for a study protocol under a higher PIP.

Here, increasing the PIP resulted in SVV elevation. In addition, the positive correlation between hemorrhage and SVV had significant interactive effects with increasing PIP. This indicates that a hypovolemic state enhanced the effects of PIP on SVV.

These observations can be explained by the mechanisms of SVV. A maximal SV (SVmax) is a measure of the inspiratory elevation of the left ventricle (LV) SV [20–22]. Mesquida et al. [23] demonstrated that intrathoracic blood volume, calculated as the difference between the right ventricle and LV SV, gradually increases in the expiratory phase, and decreases in the inspiratory phase of mechanical ventilation. This filling of the intrathoracic blood volume in the expiratory phase temporarily reduces LV preload and results in a minimal SV (SVmin) at the beginning of expiration. Hence, SVmin indicates the expiratory fall of the SV. Preisman et al. [24] clearly indicated that increasing the Vt reduces the pulse pressure at the beginning of expiration. In the late expiratory phase, however, SV remains stable [25], i.e., the reference SV (SVref) is maintained.

**Fig. 3:**
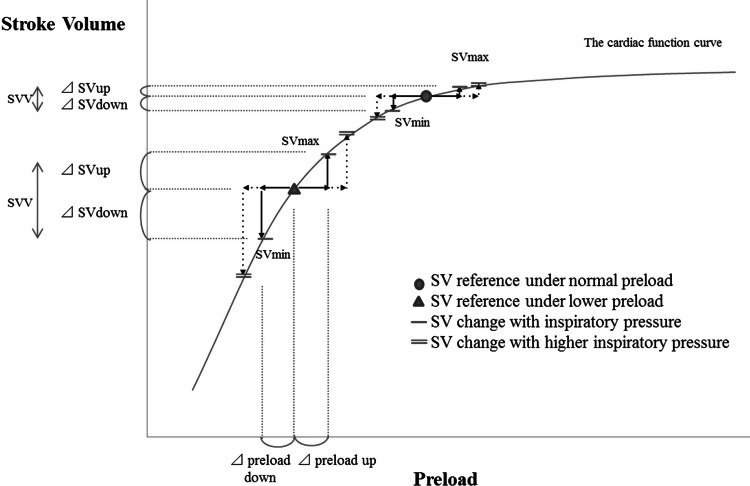
The concept of stroke volume variation (SVV) explained on the cardiac function curve. This figure illustrates that the determinants of SVV are cardiac function, preload, and changes in the preload of the left ventricle with the respiratory cycle. When preload is at normal levels (*circle*), delta stroke volume up (ΔSVup) and delta stroke volume down (ΔSVdown), generated by respiration-induced changes in the preload of the left ventricle, are small. When preload reduces and shifts to the triangle, however, a large difference between the maximal stroke volume (SVmax) and minimal stroke volume (SVmin) is observed, which indicates increased SVV in comparison with a normal preload state. In addition, a higher inspiratory pressure can increase both the ΔSVup and ΔSVdown, resulting in an increased SVV, the effect being larger in the lower preload state than the normal preload state

Figure 3 shows how SVV is affected by PIP and preload, and substantiates the evidence provided by Renner et al. [26] that SVV at a lower Vt is not sensitive to loss of preload, and that use of SVV as a guide to fluid management at a higher Vt can lead to volume overloading.

Based on our investigation of the correlation between SVV and PIP, SVV can be indexed to PIP. Indeed, this substantiates the reports by Vistisen et al. [9–11] that indexed dynamic parameters improve the accuracy for prediction of fluid responsiveness at lower Vt. We also investigated the correlation between indexed SVV and PIP under various preload conditions. Elevation in SVV with increasing PIP was eliminated by indexation with a PIP of ≥9 cmH_2_O. However, the indexed SVV was significantly larger with a PIP of 5 cmH_2_O than with a higher PIP. This may indicate that there is a limit to indexing SVV to PIP under the conditions of low PIP, which means low Vt ventilation.

There are several limitations to the current study. First, it was performed in dogs; however, previous studies on SVV extracted using the pulse contour technique were also conducted in dogs [27–29]. Vascular responsiveness, lung compliance, relative volume of pulmonary blood flow, responses to the effects of respiratory changes, and pulse contour changes during tachycardia may differ between humans and dogs. The lung compliance of dogs is so high that their Vt reached >40 ml/kg for the maximum PIP of 21 cmH_2_O. However, we adopted the current study protocol as a higher PIP is needed for the prefixed Vt when lung compliance is low, e.g., in the case of ARDS and for the high Vt ventilation in cervical injury patients. Second, we only investigated up to the level of moderate hemorrhage of 20 ml/kg of blood, which is a safe volume of blood donation for the animals. Therefore, because we planned a non-lethal animal experiment, we could not evaluate these parameters in a model of severe blood loss. Third, we measured SV using the thermodilution method. Since the observed SVtd and MAP express the averaged values for preload rather than the beat-to-beat values for LV output, SVtd and MAP did not decrease with increasing PIP. This contrasts with the results of Morgan et al. [30], who demonstrated that increasing airway pressure and the inspiratory/expiratory ratio, decreases SV in expiration. Finally, changing PIP without PEEP surely affects end-tidal CO_2_, which can affect the SVV by changing pulmonary and systemic vascular tonus, although Kubitz et al. [31] verified that SVV is not affected by changes in cardiac afterload. To improve the clinical utility of SVV, further studies are required to reveal the precise relationship between respiratory change of the LV preload and effects of other ventilator settings.

In conclusion, we found that SVV correlates with PIP, a determinant of Vt, even under hypovolemic conditions, and that the increase in SVV induced by PIP is greater in cases of decreased preload. The indexed SVV was less susceptible to higher inspiratory pressures and should be a better parameter to evaluate fluid responsiveness under such conditions.
